# Analytical evaluation of a BNP assay on the new point-of-care platform respons^®^IQ

**DOI:** 10.1016/j.plabm.2015.04.002

**Published:** 2015-05-01

**Authors:** Stephan Fellner, Simone Hentze, Uwe Kempin, Evelyn Richter, Jörg Rocktäschel, Barbara Langer

**Affiliations:** pes Gesellschaft für medizinische Diagnosesysteme mbH (pes diagnosesysteme), Hauptstrasse 103, D-04416 Markkleeberg, Germany

**Keywords:** BNP, B-type natriuretic peptide, Point of care, Immunoassay

## Abstract

a) Objectives: respons^®^IQ is a new point-of-care (POC) immunoassay platform utilizing evanescent field total internal reflection fluorescence (TIRF) detection and active microfluidics controlled by optical sensors. A B-type natriuretic peptide (BNP) assay was developed on this system. The objective was to show that the BNP test fulfils the basic requirements regarding analytical performance, storage stability of cartridges and correlation to reference systems to be used as a POC test.

b) Design and methods: Analytical sensitivity and imprecision were determined in 10 separate experiments over a period of one year. Cartridge storage stability at 4–7 °C and 37 °C was tested. The correlation of responsIQ whole blood measurements to a POC reference device and a laboratory analyzer was determined using 100 patient samples.

c) Results: Limit of detection (LOD) was 2.3±1.0 pg/ml BNP and within-run coefficient of variation (within-run CV) was 4.8±1.4% down to a concentration of <40 pg/ml BNP. Cartridge storage stability at 4–7 °C was greater than 50 weeks and at 37 °C, stability was three weeks. The correlation of responsIQ results with both reference methods was high (*r*≥0.972).

d) Conclusions: The developed BNP test fulfils the basic requirements for the performance parameters defined above. The test׳s sensitivity was in the performance range of laboratory analyzer BNP tests. This is the first extensive proof of concept of the responsIQ system.

## Highlights

• New point-of-care platform with micro-fluidics and evanescence field technology.
• A B-type natriuretic peptide assay was validated on the system.
• Correlation with a POC and a laboratory system was high (*r*≥0.972).
• Sensitivity was in the performance range of lab analyzer tests.

## 1 Introduction

Over the last two decades, many research projects have sought to generate new analytical sensor devices applying microfluidic sample processing. Despite the diversity of the newly developed test systems only a very small number of devices have made the way to the market in the field of point-of-care (POC) immunoassay systems [1]. For applications in which quantification and high sensitivity are less important, the lateral flow test is still the tool of choice due to its simplicity and low production cost [1]. On the other hand, devices for applications with high demands regarding sensitivity and accurate quantification cannot yet match the performance of laboratory analyzers.

pes diagnosesysteme has developed a microfluidic device for POC diagnostics with active fluidics and total internal reflection fluorescence (TIRF) detection. The device consists of a single-use cartridge, which contains all biomaterials, and an instrument, which pneumatically moves the liquid, controls the microfluidic assay steps with the aid of optical sensors and reads the TIRF assay. The responsIQ is to our knowledge the only POC system that controls the fluidic action by optical sensors, which detect the position of the sample within the cartridge. These optical sensors also control the sample volume, which is loaded on the cartridge by means of a 50 µL positive displacement pipette.

As proof of concept, a BNP test has been installed on the system. BNP is a clinical marker for the diagnosis of heart failure and for risk assessment in cases of acute coronary syndrome. As BNP is present in human plasma only in low pg/mL up to low ng/mL levels, a highly sensitive test is required. The use of a typical cut-off concentration in clinical practice of 100 pg/mL for patients presenting with acute onset or worsening of symptoms or alternatively of 35 pg/mL for patients with non-acute presentations [2] requires a quantitative assay with high precision at the decision cut-offs.

The objective of this study was to show that the BNP test fulfils the basic requirements regarding analytical performance, storage stability of its cartridges and correlation to reference systems to be used as POC test. Therefore, determination of detection limit and imprecision, a study on cartridge storage stability and a comparison to reference systems using patient samples were performed.

## 2 Material and methods

### 2.1 Materials

A pair of commercially available sandwich murine anti-BNP monoclonal antibodies (mAb) was used for BNP detection. The anti-BNP clone 50E1 from Hytest Ltd (Turku, Finland) was used as capture antibody. The anti-BNP clone 24C5, also from Hytest Ltd, was used as detection antibody.

The activated cyanine dye S 0458 (CAS-No 661465-58-3) was purchased from FEW Chemicals GmbH (Bitterfeld-Wolfen, Germany).

Glycosylated proBNP was obtained from Hytest Ltd. Dry bags Minipax and Alu-PE pouches were ordered from Ströbel (Langenzenn, Germany).

Gilson Microman pipettes with fixed volume of 50 µL (custom-made adaption) and tips were made by Gilson S.A.S. (Villiers-le-Bel, France).

RFID tags MiniTrack Paper Tag 3002077 were ordered from Smarttrac Technology Group (Frankfurt am Main, Germany).

All other chemicals were from Sigma-Aldrich Chemie GmbH (Munich, Germany).

### 2.2 Samples

EDTA-anticoagulated whole blood from patients was received from a local cardiologist. These specimens were left over from routine checkups of the patients and all samples were anonymised.

EDTA plasma was generated by centrifugation of whole blood samples at 2830 g for 10 min and collection of the supernatant.

### 2.3 responsIQ – system

**Fig. 1:**
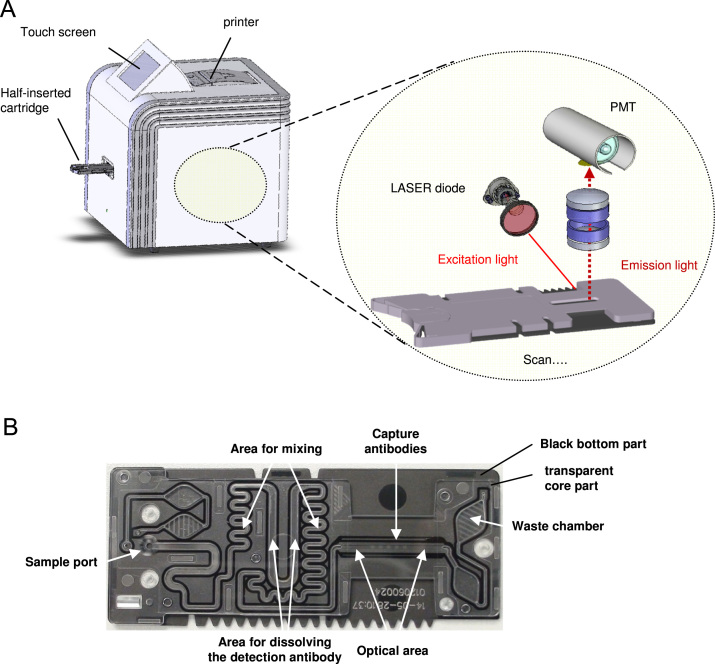
(A) responsIQ instrument and visualization of detection unit and (B) top view of cartridge without the cover part showing the microfluidic design.

responsIQ is a new POC immunoassay platform and comprises a readout instrument and ready-to-use cartridges (Fig. 1A). The platform requires 50 µL of sample (either whole blood or plasma). Measurement is performed in less than 10 min.

Several safety features, such as integrated control of sample volume and flow as well as storage of calibration and lot specific data on each cartridge, are applied.

responsIQ measures the rate of increase of the fluorescence signal, which is proportional to the concentration of analyte in solution. The resulting signal slope in V/s is read off the calibration curve by the analysis software and the BNP result is displayed on the instrument׳s screen.

Further background information on the responsIQ has been published in a patent application [3]. The measurement principle of the responsIQ has already been described by Rascher et al. [4], who have developed a procalcitonin assay on the system (Video A1).

**Video A1:**
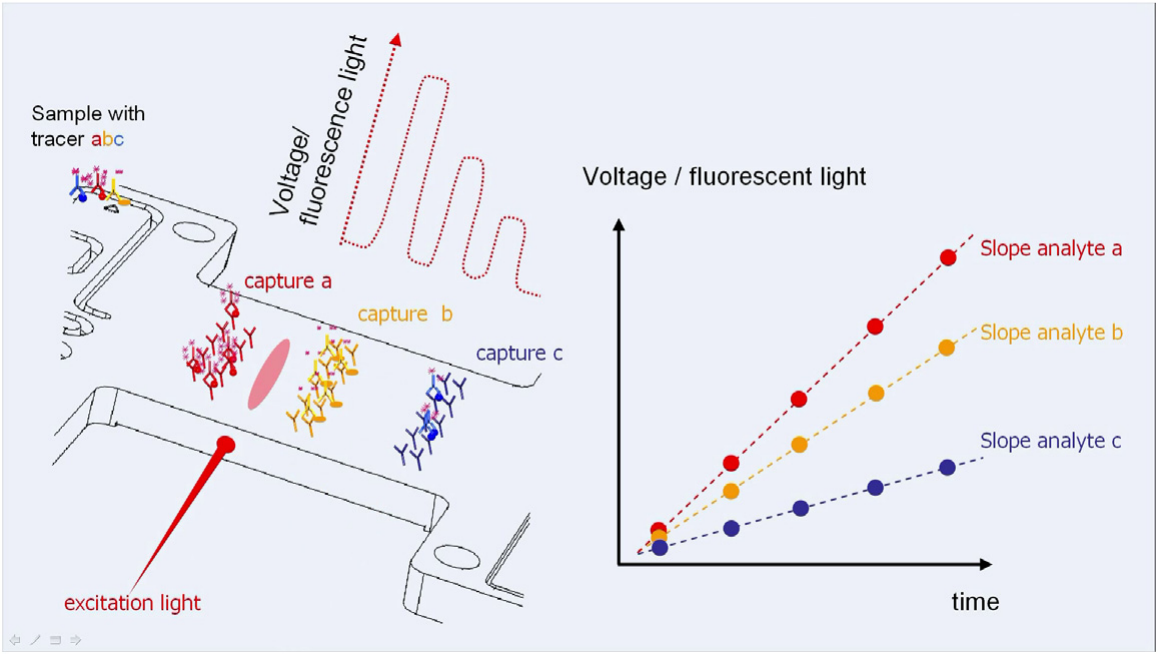
Schematic demonstration of signal generation process during a responsIQ measurement on the example of an assay for the determination of three analytes. A laser scans the detection area and the increase of the fluorescence signal over time is monitored.

#### 2.3.1 Cartridge design and production

The single-use cartridge contains a micro-fluidic system with an optical area for detection (Fig. 1B). The integrated assay reagents consist of a BNP sandwich assay. Within the measuring cell, three lines of BNP capture antibody are immobilized.

The anti-BNP detection antibody was labeled in-house with the activated cyanine dye S 0458 (*λ*_ex,max_=647 nm/*λ*_em,max_=664 nm, 2–3.5 mol dye per mol antibody) to form the BNP detection conjugate (detection antibody). The detection solution contains the detection antibody as well as buffer components and blocking components. The detection solution is dispensed into the detection antibody zone (Fig. 1B).

All assay reagents are dried into the channels for stability reasons. An integrated RFID-tag stores test- and lot-specific data. The cartridge is packaged in Alu-PE pouch with a silica gel pack.

#### 2.3.2 Calibration of responsIQ cartridges

A low endogenous EDTA plasma pool was generated by selecting and mixing samples with BNP concentration less than 15 pg/mL. The BNP concentration of the pool was measured on a Siemens ADVIA Centaur^®^ (Siemens Healthcare Diagnostics, Erlangen, Germany) and the pool was stored at −80 °C in aliquots.

Glycosylated proBNP, which is the prohormone of BNP and comprises the BNP peptide chain, was used for calibration, as it is considerably more stable towards degradation in plasma matrices than BNP [5]. Also, glycosylated proBNP has been shown to be the major component of immunodiagnostically detected BNP in patients with heart failure [6]. The manufacturer of the glycosylated proBNP (Hytest Ltd) determines the antigen mass of glycosylated proBNP with respect to the peptide content of the molecule (*M*=11905.5 g/mol). The BNP molecule has a lower molecular weight of 3464 g/mol. The concentration of a calibrator (in pg/mL) is labeled with respect to the BNP fraction of the calibrator, termed ‘BNP equivalent’ in this work, and translates to a 3.44 times higher mass of contained glycosylated proBNP.

For calibration of a new cartridge lot, the following concentrations were spiked into a low endogenous EDTA plasma pool and each calibrator was measured 3 times (see also Section 3.1):

− for low concentration calibration curve: 0, 70, 150, 270, 400 pg/mL BNP equivalents and
− for high concentration calibration curve: 700, 1300, 1900, 2500 pg/mL BNP equivalents.

**Fig. 2:**
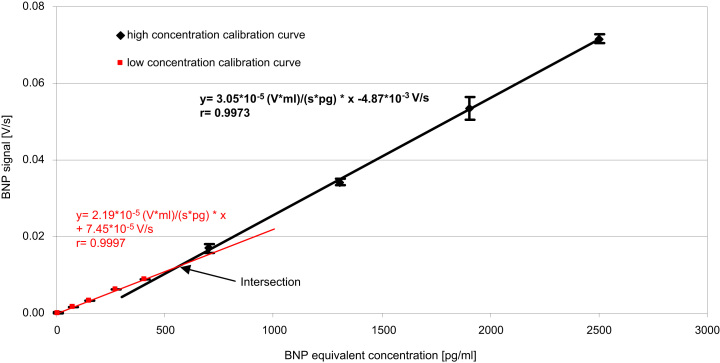
Typical calibration curve comprising two sections with different slopes according to the low and high concentration range. *N*=3. Mean values; whiskers represent standard deviation.

The calibration curves were generated by least squares fit. The intersection of both curves was calculated and used as the switch point for the domain of calibration (Fig. 2). The calibration data was written to the RFID chips of the remaining cartridges of the production lot. Afterwards the cartridges were packaged into Alu-PE pouches containing dry packs.

#### 2.3.3 Measurement

50 µL of sample (either EDTA whole blood or EDTA plasma) was loaded on the sample port of the cartridge by a Gilson Microman pipette. The cartridge lid was closed and the cartridge inserted into the responsIQ instrument. The cartridge was automatically processed by the instrument and the used cartridge ejected automatically after measurement. The BNP result was displayed on the instrument׳s screen and could be printed on the integrated printer.

### 2.4 Calculations and statistics

The limit of detection (LOD) was calculated by dividing two standard deviations of the low endogenous plasma pool measurements by the slope of the dose-response curve.

The statistical significance of differences between two groups was evaluated using Student׳s unpaired *t*-test. Values of *p*<0.05 were considered significant.

For method comparison, Passing & Bablok fit was used. Correlation coefficients were calculated using least squares fit.

## 3 Results and discussion

### 3.1 Reportable range and calibration curve

The reportable range of the responsIQ BNP test was 5–2500 pg/mL BNP. The BNP signal rises steadily with concentration in this range, but the slope of the dose-response curve is lower at the low concentration levels. To account for this non-linear behavior, two split linear regressions, one for the low concentration range and one for the high concentration range, were fitted to the respective data (Fig. 2). The slope of the linear regression for the low concentration range was typically 25% lower than the slope of the regression line for the high concentration range.

Linearity within each calibration domain was good with high correlation coefficients. The quality of the calibration curve at low concentrations is especially important as the diagnostic decision values are located in this domain.

### 3.2 Limit of detection and imprecision

In order to analytically validate and characterize the responsIQ BNP test, the LOD and the within-run coefficient of variation (within-run CV) were determined in ten separate runs over a period of about one year. Each run consisted of ten determinations of low endogenous BNP plasma pool (BNP concentrations from 3.5 to 7.8 pg/mL BNP as determined on ADVIA Centaur) as well as five determinations of each calibrator with 30 pg/mL, 60 pg/mL and 90 pg/mL BNP equivalents spiked into the respective low endogenous BNP plasma pool. Nine different cartridge lots and five different instruments were included in this experiment as indicated in Table 1.

**Table 1:** Limits of detection (LOD) and within-run coefficient of variation (CV) from 10 LOD experiments measured over a period of about one year.

Experiment no.	Date of measurement	Cartridge lot	Instrument no.	LOD [pg/mL]	Within-run CV at		
					+30 pg/ml BNP equivalents (*n*=5)	+60 pg/ml BNP equivalents (*n*=5)	+90 pg/ml BNP equivalents (*n*=5)
1	28 Jan 2013	A	1	1.9	7.6%	3.4%	5.7%
2	07 Feb 2013	B	1	1.9	3.9%	2.7%	4.4%
3	22 Mar 2013	C	1	1.9	4.4%	4.5%	4.2%
4	02 Mai 2013	D	2	2.5	2.6%	11.5%	3.4%
5	17 Jun 2013	E	3	4	6.1%	5.2%	5.9%
6	18 Jun 2013	F	3	4.3	5.0%	2.3%	6.5%
7	03 Sep 2013	G	2	1.8	6.0%	4.4%	3.6%
8	30 Oct 2013	H	4	1.6	4.0%	5.8%	4.8%
9	13 Feb 2014	I	1	1.4	4.3%	2.9%	4.5%
10	13 Feb 2014	I	5	1.8	3.7%	3.1%	1.9%
**Mean value±standard deviation:**				**2.3±1.0**	**4.8±1.4%**	**4.6±2.7%**	**4.5±1.4%**

The resulting LODs and within-run CVs are shown in Table 1.

The data show that the responsIQ BNP test is sufficiently sensitive for the use as clinical BNP test. The LOD is very low for a POC system and is comparable to the sensitivity of laboratory analyzer BNP tests [7], [8].

A constructional difference of the responsIQ cartridge compared to most POC devices might explain its high sensitivity for analytes that tend to adsorb to surfaces. The responsIQ cartridge contains no porous materials or membranes, which would display a high surface area to the sample. Most POC devices contain such high-surface-area elements, which can potentially lead to adsorption of analyte molecules to this surface and as a consequence to reduced sensitivity. Highly charged proteins, such as BNP or Troponin I, tend to adsorb to surfaces [9]. BNP has an isoelectric point of 10.95 [10] and Troponin I an isoelectric point of 9.9 [11], which leads to highly charged species of these two analytes at neutral pH.

### 3.3 Shelf life and accelerated temperature storage

**Fig. 3:**
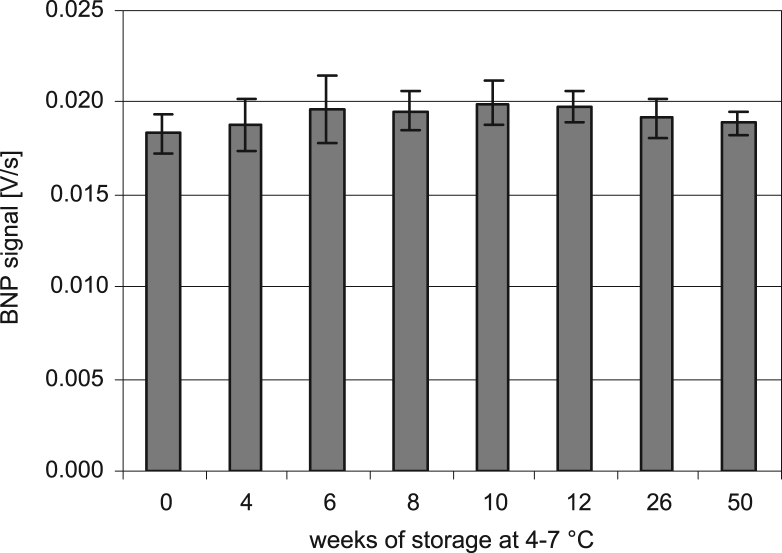
Shelf life of responsIQ BNP cartridges stored at 4–7 °C. Bars represent mean values of BNP signals generated on cartridges by goat serum containing 1000 pg/ml BNP equivalents after storage as indicated. Whiskers represent standard deviation. *N*=6–8.

The shelf life of the cartridge unit at 4–7 °C was assessed. A cartridge lot was produced and stored at 4–7 °C. Cartridges were tested with goat serum containing 1000 pg/mL BNP equivalents on the first day following production as well as on subsequent measurement days up to 50 weeks following production using the same instrument throughout the complete study. The data is displayed in Fig. 3. It shows that even after 50 weeks of storage at 4–7 °C no significant reduction of the BNP signal could be observed. Thus, a cartridge shelf life of 50 weeks or greater could be demonstrated.

For accelerated temperature testing, cartridges were stored at 37 °C for 3 weeks starting one week following production. Cartridges were measured with goat serum containing 1000 pg/mL BNP equivalents (data not shown). For comparison, cartridges from the same lot, which were stored for the entire period at 4 °C, were measured in the same way. There was no significant change in the assay signal when both storage groups were compared (signal difference: 1%, each group *N*=6, *p*=0.744). Thus the cartridges can be considered to be stable at 37 °C for at least 3 weeks.

### 3.4 Comparison to reference systems

A comparison to reference systems using patient samples was carried out. The goal was to determine the correlation of responsIQ whole blood measurements with an established POC device (Alere Triage^®^ BNP, Alere Inc., Waltham, MA, USA) as well as with an established laboratory analyzer device (Siemens ADVIA Centaur^®^ BNP, Siemens Healthcare Diagnostics). One responsIQ instrument was installed at a local cardiologist׳s office. There, leftover EDTA/whole blood samples were measured on the responsIQ BNP test (single determination) on-site by nurses in parallel to the routine Triage BNP test. In total, 100 patient samples were analyzed over a period of about 8 weeks without any pre-selection of the samples. The hematocrit of all samples was determined (mean value: 41%, range: 22–51%).

**Fig. 4:**
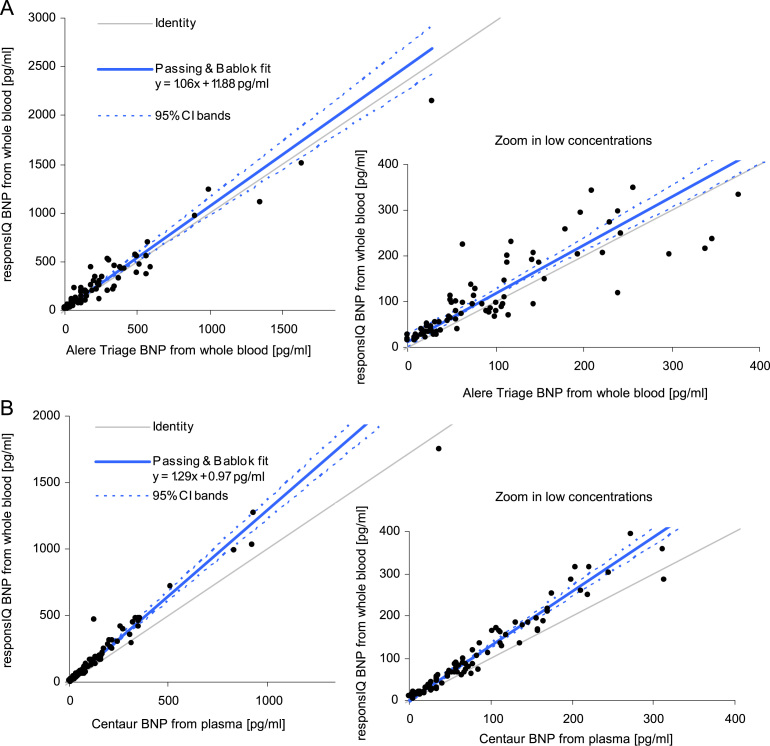
Passing & Bablok fit of responsIQ BNP values from whole blood (A) versus Alere Triage BNP also from whole blood; both values measured on-site (single measurement) directly after blood draw. *N*=100; and (B) versus ADVIA Centaur BNP values from plasma. responsIQ double determination in pes diagnosesysteme lab and Centaur BNP single measurement in external lab. *n*=98.

Comparison of responsIQ BNP values to Triage BNP is displayed in Fig. 4A. The analyzers show good overall correlation (*r*=0.972). The slope of 1.06 shows that, on average, both systems give comparable results.

For comparison to the laboratory analyzer ADVIA Centaur BNP the 100 samples described above were transferred to the laboratory of pes diagnosesysteme one to five hours after blood draw. Within the following hour, whole blood samples and plasma samples were measured in duplicate on the responsIQ. The remaining plasma was frozen at −80 °C immediately afterwards and sent to a clinical lab for measurement on the Centaur BNP test later. The reason for the second determination of the whole blood samples on the responsIQ for comparison to Centaur BNP was the low storage stability of BNP in whole blood samples at room temperature. Two samples were excluded from Centaur measurement because these had not been stored as specified.

Comparison of responsIQ BNP whole blood measurements with the laboratory analyzer test Centaur BNP is displayed in Fig. 4B. Again, a strong correlation can be observed with *r*=0.974. The slope of 1.29 indicates that results determined by the responsIQ are typically higher than the Centaur BNP results. The intercept of the regression line is negligible.

The results show that the responsIQ BNP measurements from whole blood correlate to both reference devices used in this study. From Fig. 4 it can be seen, that responsIQ BNP values show less variation from the regression line when correlated to the lab analyzer ADVIA Centaur BNP than to Alere Triage BNP. The tighter correlation to the Centaur BNP might be in part due to similar epitopes of the antibody pairs used in Centaur BNP and responsIQ BNP assays as well as the duplicate measurement which was carried out on the responsIQ in this comparison.

The results from whole blood and plasma both measured on the responsIQ were also compared (data not shown) and show high correlation (*r*=0.998). The Passing and Bablok fit resulted in a slope of 0.89 and an intercept of 5.44 pg/mL BNP. Thus, whole blood result values were on average 11% lower than the related plasma results.

## 4 Conclusions

In this study, the performance parameters limit of detection, imprecision, storage stability of cartridges and correlation to reference systems were characterized. It was shown that the responsIQ is a suitable device for the quantitative analysis of BNP from clinical samples. Also, the basic requirements for cartridge storage stability could be met. The sensitivity was in the performance range of laboratory analyzer tests. In conjunction with the safety features installed on the system, the responsIQ could help make POC analysis safer.

This is the first extensive validation of an assay on the responsIQ system.

## References

[1] Chin C.D., Linder V., Sia S.K. (2012). Commercialization of microfluidic point-of-care diagnostic devices. Lab Chip.

[2] McMurray J.J., Adamopoulos S., Anker S.D., Auricchio A., Böhm M., Dickstein K. (2012). The task force for the diagnosis and treatment of acute and chronic heart failure 2012 of the European Society of Cardiology. ESC Guidelines for the diagnosis and treatment of acute and chronic heart failure 2012. Eur Heart J.

[3] Schenk R, inventor; DiaSys Diagnostic Systems GmbH, assignee. Messkassette und Messvorrichtung für die Detektion von Zielmolekülen in einer flüssigen Probe durch Messung von Fluoreszenzemission nach Anregung im evaneszenten Feld. German patent application DE102010038431. January 26; 2012.

[4] Rascher D., Geerlof A., Kremmer E., Krämer P., Michael S., Hartmann A. (2014). Total internal reflection (TIRF)-based quantification of procalcitonin for sepsis diagnosis – a point-of-care testing application. Biosens Bioelectron.

[5] Niederkofler E.E., Kiernan U.A., O’Rear J., Menon S., Saghir S., Protter A.A. (2008). Detection of endogenous B-type natriuretic peptide at very low concentrations in patients with heart failure. Circ Heart Fail.

[6] Seferian K.R., Tamm N.N., Semenov A.G., Mukharyamova K.S., Tolstaya A.A., Koshkina E.V. (2007). The brain natriuretic peptide (BNP) precursor is the major immunoreactive form of BNP in patients with heart failure. Clin Chem.

[7] Abbott Diagnostics Division. Package insert: Architect BNP on Abbott Architect iSystems. Abbott park. IL: Abbott Diagnostics Division, 260-737 10/08; 2008 Nov. 〈http://www.ilexmedical.com/files/PDF/BNP_ARC.pdf〉 [accessed 08.01.2015].

[8] Siemens Healthcare Diagnostics. Instructions for use: BNP on the Siemens Centaur/Centaur XP; 10629823_EN Rev. P, 2011-07.

[9] Buechler KF, McPherson PH, Inventors; Biosite Inc, assignee. Methods for improving the recovery of troponin I and T in membranes, filters and vessels. United States patent US6627404B1; 2003. September 30.

[10] Hytest Ltd. Hytest Technotes: Human proBNP and proBNP derived peptides (BNP and NT-proBNP) 2010 August. https://www.hytest.fi/sites/52cd5c487653512f63000004/content_entry52cd6295765351528d000020/52cd6298765351528d000051/files/Human_ProBNP_and_proBNP-derived_peptides_TechNotes.pdf [accessed 02.12.2014].

[11] Filatov V.L., Katrukha A.G., Bulargina T.V., Gusev N.B. (1999). Troponin: structure, properties, and mechanism of functioning. Biochemistry.

